# Melatonin-Induced Protective Effects on Cardiomyocytes Against Reperfusion Injury Partly Through Modulation of IP3R and SERCA2a Via Activation of ERK1

**DOI:** 10.5935/abc.20180008

**Published:** 2018-01

**Authors:** Shunying Hu, Pingjun Zhu, Hao Zhou, Ying Zhang, Yundai Chen

**Affiliations:** Chinese PLA General Hospital, Beijing - China

**Keywords:** Melatonin, Myocardial Reperfusion, Cardiac Myocytes, Myocardial Infarction, Heart Failure

## Abstract

**Background:**

Melatonin is a neuroendocrine hormone synthesized primarily by the pineal gland that is indicated to effectively prevent myocardial reperfusion injury. It is unclear whether melatonin protects cardiac function from reperfusion injury by modulating intracellular calcium homeostasis.

**Objective:**

Demonstrate that melatonin protect against myocardial reperfusion injury through modulating IP3R and SERCA2a to maintain calcium homeostasis via activation of ERK1 in cardiomyocytes.

**Methods:**

In vitro experiments were performed using H9C2 cells undergoing simulative hypoxia/reoxygenation (H/R) induction. Expression level of ERK1, IP3R and SERCA2a were assessed by Western Blots. Cardiomyocytes apoptosis was detected by TUNEL. Phalloidin-staining was used to assess alteration of actin filament organization of cardiomyocytes. Fura-2 /AM was used to measure intracellular Ca^2+^ concentration. Performing in vivo experiments, myocardial expression of IP3R and SERCA2a were detected by immunofluorescence staining using myocardial ischemia/ reperfusion (I/R) model in rats.

**Results:**

In vitro results showed that melatonin induces ERK1 activation in cardiomyocytes against H/R which was inhibited by PD98059 (ERK1 inhibitor). The results showed melatonin inhibit apoptosis of cardiomyocytes and improve actin filament organization in cardiomyocytes against H/R, because both could be reversed by PD98059. Melatonin was showed to reduce calcium overload, further to inhibit IP3R expression and promote SERCA2a expression via ERK1 pathway in cardiomyocytes against H/R. Melatonin induced lower IP3R and higher SERCA2a expression in myocardium that were reversed by PD98059.

**Conclusion:**

melatonin-induced cardioprotection against reperfusion injury is at least partly through modulation of IP3R and SERCA2a to maintain intracellular calcium homeostasis via activation of ERK1.

## Introduction

Myocardial ischemia-reperfusion injury typically arises in patients presenting with acute ST-segment elevation myocardial infarction (STEMI), in whom the most effective therapeutic intervention for reducing acute myocardial ischemic injury and limiting the size of myocardial infarction (MI) is timely and effective myocardial reperfusion therapy.^1^ However, the process of myocardial reperfusion can itself induce further myocardial reperfusion injury.^1-4^ Myocardial reperfusion injury weakens the benefit of reperfusion therapy and brings to patients larger MI size, more severe heart failure and worse prognosis. Restoration of cardiac circulation is accompanied by cell damages and death (lethal reperfusion injury), reperfusion arrhythmias, myocardial stunning, and no-reflow phenomenon. Lethal reperfusion injury (cardiomyocyte death induced by reperfusion) is a key therapeutic target with anticipated significant impact on the patient’s prognosis.^1-6^ Melatonin (N-acetyl-5-methoxytryptamine) is a neuroendocrine hormone, which is synthesized primarily by the pineal gland.^7,8^ Melatonin presents profound protective effects against myocardial ischemia-reperfusion injury through antioxidant actions.^9-18^ Ca^2+^ overload is the primary stimulator to ischemia/reperfusion injury and induce cardiomyocytes apoptosis in ischemia/reperfusion condition. It is unclear whether melatonin protects cardiac function from reperfusion injury by modulating intracellular calcium homeostasis. Yeung et al.^19^ suggested that melatonin is cardioprotective against chronic hypoxia-induced myocardial injury because it improves calcium handling in the sarcoplasmic reticulum (SR) of cardiomyocytes via an antioxidant mechanism.

However, the evidence about melatonin’s effect and underlying mechanism on Ca^2+^ overload under acute ischemia/reperfusion is rare. The cardiac inositol 1,4,5-trisphosphate receptors (IP3R) and sarcoplasmic reticulum Ca^2+^-ATPase (SERCA2a) are key mediators for intracellular calcium handling, contractility and death in cardiac cells.^20-23^ So in the present study we hypothesized melatonin has protective effects on cardiomyocyte against reperfusion injury through modulating IP3R and SERCA2a to maintain intracellular calcium homeostasis. Ischemia-reperfusion has been shown to activate the anti-apoptotic pro-survival kinase signalling cascades including p42/p44 extra-cellular signal-regulated kinases (ERK 1/2) which have been implicated in cellular survival.^24,25^ It is not clear if ERK1 plays important role during modulation of melatonin on calcium homeostasis in cardiomyocytes. The present study aimed to elucidate whether melatonin protects cardiomyocytes against reperfusion injury through modulating IP3R and SERCA2a to reduce calcium overload via ERK1 pathway.

## Methods

### Ethics statement

The present study was performed in accordance with the guidelines of the Ethic Committee of Chinese PLA (People's Liberation Army) General Hospital, Beijing, China.

### H9C2 Cells culture

H9C2 cells (derived from the rat embryonic cardiomyoblast) were obtained from Chinese Academy of Medical Sciences (Shanghai, China) were cultured in Dulbecco’s Modified Eagle Medium: Nutrient Mixture F-12 (DMEM/F12, Thermo Fisher Biochemical Products, Beijing, China) supplemented with 10% fetal bovine serum (FBS, Invitrogen Life Technologies, Carlsbad, CA, USA) and 100 mg/mL penicillin/streptomycin (Beyotime Institute of Biotechnology, China).

### H/R injury induction in vitro and melatonin or plus with PD98059 treatment

Hypoxic conditions were produced using fresh Hanks solution with 95% N_2_ and 5 % CO_2_. The pH was adjusted to 6.8 with lactate to mimic ischemic conditions. The dishes were put into a hypoxic incubator (Invivo2-400, Ruskinn) that was equilibrated with 95% N_2_ and 5% CO_2_and the actual oxygen concentration was zero. Ambient O_2_ levels in the hypoxia incubator were monitored by an O_2_ analyzer (series-2000, Alpha Omega Instruments). After hypoxic treatment, the culture medium was rapidly replaced with fresh DMEM with 1% FBS to initiate reoxygenation. Hypoxia/reoxygenation procedure was achieved by 4 h of hypoxia treatment (anoxia) and 4 h of reoxygenation treatment. For melatonin treatment, cultured cells were pre-incubated with melatonin (5 uM) 12 h before hypoxia, or plus with PD98059 with concentration of 10 uM prior to melatonin treatment. The dose of melatonin was chosen according to previous studies.^18,26^

### In vitro TUNEL apoptosis assay of cardiomyocytes by confocal microscopy

The apoptosis of H9C2 cells was examined by TUNEL assay. Briefly, cultured cardiomyocytes were fixed with 4% paraformaldehyde (PFA) (Millipore, USA) and permeabilized with 1% Triton X-100 (Sigma Aldrich, USA) in phosphate-buffered saline (PBS) (Invitrogen, USA) for 30 minutes, followed by 3 times (3×10 mins) wash with fresh PBS. Then, an Apo-BrdU in Situ DNA Fragmentation Assay Kit (BioVision, USA) was applied for 1 hour, followed by incubating the treated plates with 5 µl anti-BrdUFITC antibody. Fifteen minutes of DAPI immunostaining were performed to identify the nuclei of cardiomyocytes. Then, the images were taken with an inverted Leica TCS-SP2 AOBS confocal laser-scanning microscope (Leica, Germany). Apoptosis was quantified as the percentage of cultured cardiomyocytes.

### F-actin study with fluorescent phalloidin and confocal microscopy

F-actin detection with phalloidin was done according to manufacturer’s instructions. Briefly, H9C2 were fixed on polylysine-treated glass with 3.7% paraformaldehyde and later washed with 0.1% Triton X-100-PBS. Thereafter they were stained with 0.8unit/ml fluorescent FITC-phalloidin conjugate solution (KeyGen Bio TECH Corp,China) for 10 min at room temperature. Finally, they were washed three times with PBS. Mounted samples were analyzed using confocal microscopy.

### Detection of intracellular Ca^2 +^ concentration

Intracellular Ca^2+^ was measured using the calcium-dependent fluorescent dye Fura-2 according to the manufacturer’s instructions. Briefly, H9C2 cultures were transferred to 1 mL fresh DMEM containing 5 µL Fura-2-acetoxy-methylester (AM; 10 µM; Life Technologies, Carlsbad, CA, USA) and incubated in a CO_2_ incubator at 37ºC for 1 h. Fura-2-loaded cells were then placed on the stage of a confocal microscopy (Olympus) and viewed using a 60× oil immersion objective.

### Western blots

Following the appropriate treatments, cultured cells were lysed with RIPA lysis buffer (Beyotime,China) for 30 min and centrifuged at 14,000xg for 30 min. Equal amounts of protein were separated by 10% sodium dodecyl sulfate-polyacrylamide gel electrophoresis and transferred to a polyvinylidene difluoride membrane (Millipore). After being blocked with 5% milk in Tris buffered saline containing 0.05% Tween20 (TBST) at room temperature for 1h, the membrane was incubated at 4ºC overnight with the following primary antibodies: t-ERK1(1:2000, Abcam), p-ERK1(1:1000, Abcam), IP3R (1:1000,Abcam), and SERCA2a (1:1000,Ab-cam). After being washed with TBST and further incubated with the appropriate secondary antibody at 37ºC for 60 min, the blots were visualized with an enhanced chemiluminescence (ECL) reagent.

### Myocardial ischemia/reperfusion (I/R) model and melatonin treatment

Male Sprague-Dawley (SD) rats (250 ± 10 g) were purchased from the Experimental Animal Center, Chinese PLA General Hospital. All procedures were approved by the Institutional Animal Care and Use Committee of the Chinese PLA General Hospital. Rats were divided into the following groups (n = 5 in each group): (1) Control group, (2) I/R group, (3) I/R+Melatonin group, (4) I/R+Melatonin+PD98059. Rats were intraperitoneally anaesthetized with sodium pentobarbital (50 mg/kg). The animals were then incubated and ventilated by a volume- regulated respirator during surgery. After a left lateral thoracotomy and pericardectomy, the left coronary artery was identified and gently ligated with a 6.0 prolene suture. Successful AMI was confirmed by the typical ST segment elevation in electrocardiography. Myocardial ischemia lasted for 30 mins and reperfusion for 2 hours. Freshly prepared melatonin (Sigma-Aldrich, St. Louis, MO, USA) was administered intraperitoneally at a dose of 20 mg/kg 12 hours prior to MI. PD98059 (ERK1 inhibitor, Sigma,USA) was administered with intraperitoneal injection at a dose of 5 mg/kg 4 hours prior to melatonin treatment. At the end of the reperfusion period, the hearts were excised for the preparation of sections (4 µm thickness) to detect the expression of SERCA2a and IP3R by immunofluorescence staining.

### Detection of myocardial SERCA2a and IP3R expression by immunofluorescence staining

After being treated as above, the heart sections were fixed with 4% paraformaldehyde in PBS for 15 min at room temperature, permeabilized with 0.1% Triton X-100 for 10 min, and then blocked with 5% BSA for 1 h. Then, samples were incubated overnight at 4ºC with monoclonal mouse anti-rat SERCA2a antibody (1:100;Abcam, Cambridge, USA). After being washed with PBS for three times, samples were incubated with goat anti-mouse polyclonal IgG (1:400; Abcam,Cambridge, USA) at room temperature in the dark for 2 h. For nuclear counterstaining, samples were incubated with 4’, 6-diamidino-2-phenylidone (DAPI; Sigma, USA) for 5 min. Finally, the immunofluorescence images were obtained by inverted fluorescence microscope (Olympus, Tokyo, Japan).

### Statistical analysis

Data were described as the mean ± SD of at least three independent analyzed experiments. The differences among more than 2 groups were evaluated through 1-way ANOVA (all data met the variances homogeneity and normal distribution),and LSD method was used to compare the statistical difference in the post-hoc analysis. A value of P < 0.05 was considered statistically significant. All of the statistical analyses were performed with SPSS for Windows version 16.0 (SPSS Inc., Chicago, IL).

## Results

### Melatonin promoted activation of ERK1 in cardiomyocytes against H/R

**Figure 1:**
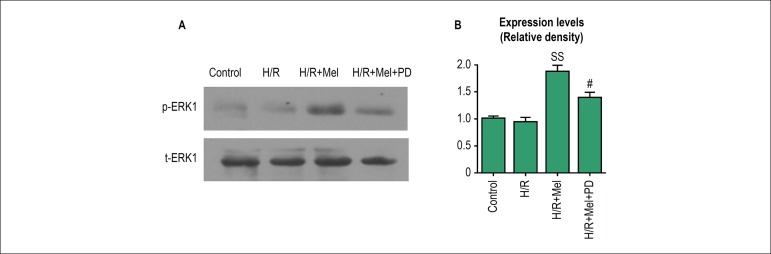
Melatonin promoted activation of ERK1 in H9C2 cells against H/R. H9C2 cells incubated in normal condition or in simulated H/R condition, in simulated H/R condition plus pretreatment with melatonin, or in simulated H/R condition plus pretreatment with melatonin and PD98059 (ERK1 inhibitor).The expression levels of phosphorylated ERK1 (p-ERK1) were evaluated by Western blotting (A) and were quantitatively analyzed (B). All values are presented as the mean±SD. n = 3. SSp < 0.01 vs. H/R group; #p < 0.05 vs. H/R+Mel group.(Control: control group; H/R:H/R group; H/R+mel: H/R+ melatonin group; H/R+mel+PD: H/R+ melatonin+PD98059 group)

At 4h after reoxygenation, we investigated the effect of melatonin on phosphorylation of ERK1 (p-ERK1) using Western blot. The expression level of p-ERK1 did not show significant difference between control and H/R group. Melatonin significantly promoted the expression of p-ERK1 in cardiomyocytes which was reversed by PD98059 (ERK1 inhibitor) (Figure 1).

### Melatonin prevents H/R-induced apoptosis of cardiomyocytes via ERK1 pathyway in vitro

**Figure 2:**
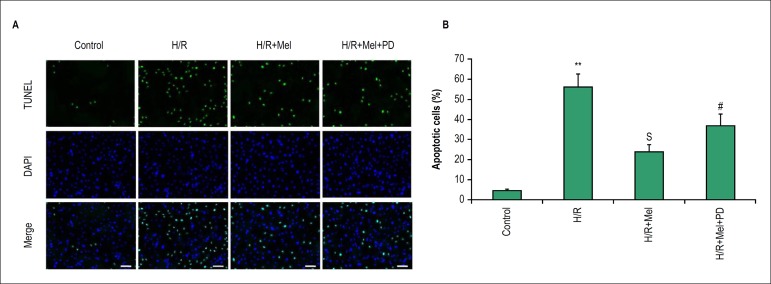
Melatonin prevents H9C2 cells apoptosis against H/R via ERK1 in vitro. Apoptosis was assessed by fluorescence TUNEL. Representative TUNEL staining images (A) and quantitative analysis in H9C2 cells(B). bar = 50 µm. All values are presented as the mean±SD. n = 3.**p < 0.01 vs. control group; Sp < 0.05 vs. H/R group; #p < 0.05 vs. H/R+Mel group. (Control: control group; H/R:H/R group; H/R+mel: H/R+ melatonin group; H/R+mel+PD: H/R+ melatonin+PD98059 group)

The apoptosis of H9C2 cells was detected at 4h after reoxygenation by TUNEL staining. The results demonstrated H/R induce apoptosis of H9C2 cells in vitro. Pretreament with melatonin decreased H/R-induced apoptosis of H9C2. The results showed percentage of apoptotic cells was obviously higher in H/R group compared to control group, however, which was significantly lower in melatonin group than H/R group. PD98059 (ERK1 inhibitor) reduced the effect of melatonin on preventing cardiomyocytes apoptosis against H/R (Figure 2).

### Melatonin protects F-actin organization in H9C2 cells against H/R via ERK1 pathway

**Figure 3:**
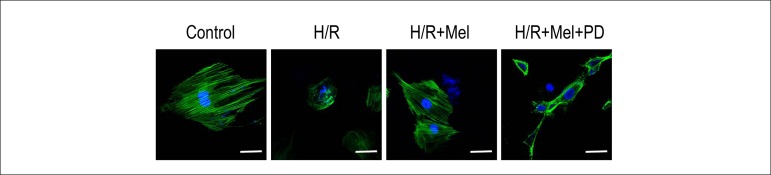
Melatonin protects F-actin organization in H9C2 cells against H/R via ERK1 in vitro. Representative confocal microscopy images show H9C2 cells stained with FITC-phalloidin. The results showed that simulated H/R induced more diffuse and irregular actin disposition compared with control group. Melatonin preserved more regular and well-defined actin organization and PD98059 (ERK1 inhibitor) reduced the protection of melatonin. bar = 20µm. (Control: control group; H/R:H/R group; H/R+mel: H/R+ melatonin group; H/R+mel+PD: H/R+ melatonin+PD98059 group)

We investigated F-actin organization in H9C2 cells at 4h after reoxygenation by fluorescent FITC-phalloidin staining. Control cardiomyocytes showed regular and well-defined actin organization, while cardiomyocytes in H/R group showed a more diffuse and irregular F-actin disposition. The differences can be visualized in the representative cardiomyocytes. Pretreatment of melatonin improved F-actin organization in cardiomyocytes compared with H/R group, but PD98059 damaged F-actin organization by inhibiting melatonin’s effect (Figure 3).

### Melatonin reduces Ca^2 +^ overload in cardiomyocytes against H/R via ERK1

**Figure 4:**
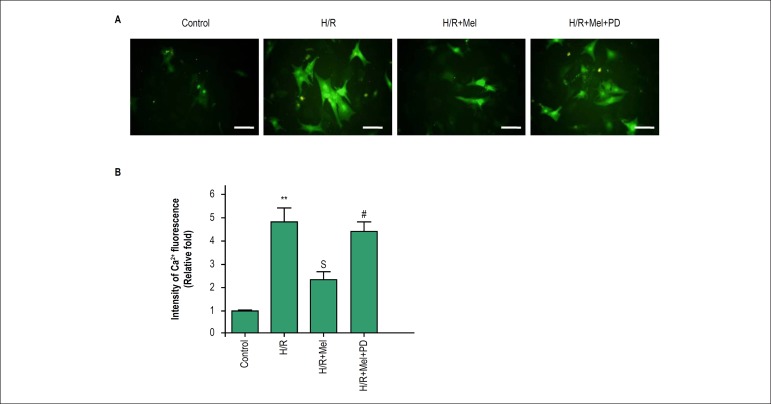
Melatonin reduces Ca^2+^ overload in H9C2 cells against H/R via ERK1 in vitro. Ca^2+^ content was assessed using Fura-2/AM in H9C2 cells incubated in normal condition or in simulated H/R condition, in simulated H/R condition plus pretreatment with melatonin, or in simulated H/R condition plus pretreatment with melatonin and PD98059 (ERK1 inhibitor). The green fluorescence intensity by Fura-2 was obviously stronger in H/R group, and melatonin pretreatment reversed the change which was inhibited by ERK1 inhibitor.bar = 30 µm. All values are presented as the mean ± SD. n = 3.**p < 0.01 vs. control group; Sp < 0.05 vs. H/R group; #p < 0.05 vs. H/R+Mel group. (Control: control group; H/R:H/R group; H/R+mel: H/R+ melatonin group; H/R+mel+PD: H/R+ melatonin+PD98059 group)

At 4h after reoxygenation, we investigated effect of melatonin on H/R-induced Ca^2+^ overload in cardiomyocytes using the calcium-dependent fluorescent dye Fura-2. The results showed the fluorescence was stronger in H/R group than in control group, meanwhile the fluorescence was decreased in melatonin group compared with H/R group, which indicated that H/R caused a marked increase of cytosolic Ca^2+^ concentration and that melatonin pretreatment significantly inhibited H/R-induced increase of cytosolic Ca^2+^ concentration which was reduced by PD98059 (Figure 4).

### Melatonin modulated expression of IP3R and SERCA2a in cardiomyocytes against H/R via ERK1

**Figure 5:**
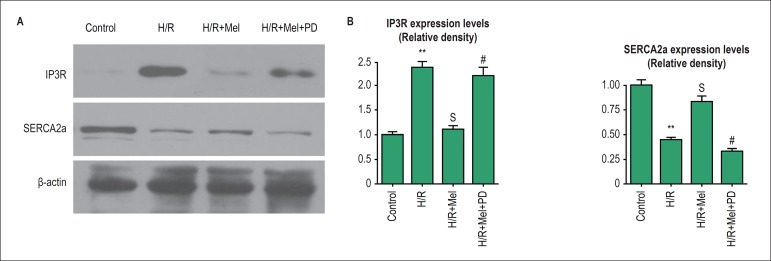
Melatonin modulated expression of SERCA2a and IP3R in H9C2 cells against H/R via ERK1 pathway in vitro. The results indicated melatonin inhibited expression of IP3R and promoted expression of SERCA2a which was reduced by PD98059. Representative Western blot images (A) and quantitative analysis (B-C) showed melatonin’s effect on expression of IP3R and SERCA2a via ERK1 pathway in H9C2 cells against H/R.bar = 30 µm. **p < 0.01 vs. control group; Sp < 0.05 vs. H/R group; #p < 0.05 vs. H/R+Mel group. (Control: control group; H/R:H/R group; H/R+mel: H/R+ melatonin group; H/R+mel+PD: H/R+ melatonin+PD98059 group)

At 4h after reoxygenation, we investigated the effect of melatonin on expression of IP3R and SERCA2a in H9C2 by Western blot. The results indicated H/R increase expression of IP3R and reduce expression of SERCA, respectively. Pretreatment of melatonin inhibited expression of IP3R and induced expression of SERCA, which were reversed by PD98059. (Figure 5).

### Melatonin modulated expression of IP3R and SERCA2a via ERK1 pathway in reperfused rat hearts

**Figure.6:**
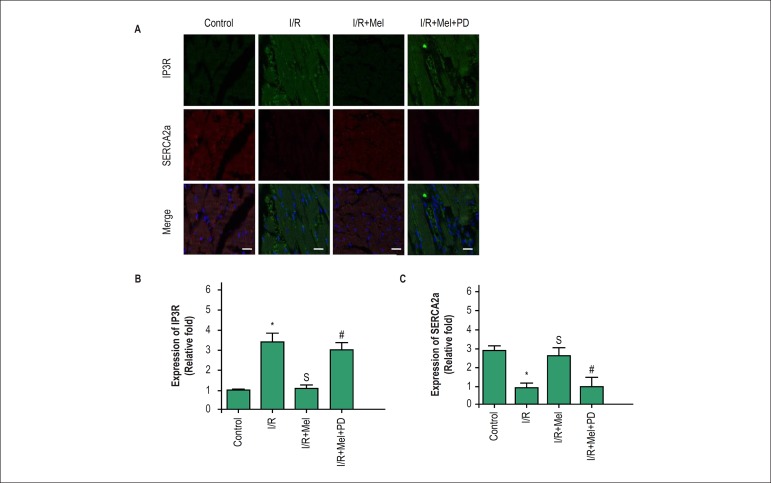
Melatonin modulated expression of IP3R and SERCA2a via ERK1 pathway in reperfused rat hearts. In reperfused myocardium, expression of IP3R and SERCA2a were assessed by immunofluorescence staining (A) and quantitative analysis (B-C). The results showed that fluorescence intensity of IP3R was increased in I/R group more than in control group, but was lower in melatonin group compared with I/R group. On the contrary, melatonin increased expression of SERCA2a in reperfused myocardium. Both of the effects of melatonin on expression of IP3R and SERCA2a were inhibited by PD98059 (ERK1 inhibitor). bar = 30 µm. All values are presented as the mean ± SD. n = 5.*p < 0.05 vs. control group; Sp < 0.05 vs. I/R group; #p < 0.05 vs. I/R+Mel group. (Control: control group; I/R:I/R group; I/R+mel: I/R+ melatonin group; I/R+mel+PD: I/R+ melatonin+PD98059 group).

In vivo, we investigated the effect of melatonin on expression of IP3R and SERCA2a in reperfused rat hearts. IP3R expression was higher in I/R group compared with control group, and melatonin reversed the change. The results demonstrated expression of SERCA2a was lower in I/R group compared with control group, but expression of SERCA2a was higher in melatonin group than I/R group. The pretreament of PD98059 reduced the effect of melatonin on expression of IP3R and SERCA2a in rat hearts against I/R (Figure 6).

## Discussion

Reperfusion-induced death of cardiomyocytes that were viable at the end of the ischemic event is defined as lethal myocardial reperfusion injury (reperfusion infarction).^1,3,27^ The existence of lethal myocardial reperfusion injury has been inferred in both experimental MI models and in patients with STEMI(1). The major contributory factors for reperfusion-induced death of cardiomyocytes include oxidative stress, calcium overload, mitochondrial permeability transition pore (mPTP) opening, and hypercontracture.^28,29^ Ca^2+^ overload is one of the main actors of this lethal reperfusion injury,^30^ which results in part from excessive sarco/endoplasmic reticulum (SR/ER) Ca^2+^ release and Ca^2+^ influx through the plasma membrane.^31^ Although ryanodine receptors (RyRs) are the major cardiac SR/ER Ca^2+^-release channels involved in excitation-contraction coupling and ischemia-reperfusion injury,^32,33^ some studies reported an increasing role for inositol 1,4,5-trisphosphate receptors (IP3R) Ca^2+^-release channels in the modulation of excitation-contraction coupling and cell death.^22,23^ Gomez et al^34^ indicated that inhibition of IP3R Ca^2+^ channeling complex limited SR/ER Ca^2+^ release and reduced both cytosolic and mitochondrial Ca^2+^ overload and inhibited subsequent PTP opening. Meantime, the cardiac SERCA2a is a key pump responsible for intracellular calcium handling and contractility in cardiac cells. Impaired calcium reuptake resulting from decreased expression and activity of SERCA2a is a hallmark of HF.^20^ IP3R and SERCA2a have been confirmed to play important roles in maintaining intracellular calcium homeostasis in cardiomyocytes.^20,22,23,35^

Melatonin as one type of neuroendocrine hormone, is synthesized primarily by the pineal gland.^7,8^ Previous studies showed melatonin confers important protective effects against myocardial ischemia-reperfusion injury.^9-14^ Melatonin administration showed to contribute to the rehabilitation of contractile function on isolated heart during reperfusion and to reduce the sensitivity of mPTP opening to Ca^2+^.^36^

Melatonin has also demonstrated to play a role in the mitochondrial adaptive changes.^37^ Melatonin and its metabolites efficiently interact with various ROS and reactive nitrogen species, and additionally they up regulate antioxidant enzymes and downregulate pro-oxidant enzymes.^9,15,16^ Previous studies confirmed that melatonin pretreatment attenuated IR injury by reducing oxidative damage and inhibiting mPTP opening. However, the evidence about melatonin’s effect and underlying mechanism on Ca^2+^ overload under acute ischemia/reperfusion is rare. The present study demonstrated that melatonin performs cardioprotection through modulation of IP3R and SERCA2a to maintain calcium homeostasis via ERK1 pathway in cardiomyocytes. ERK1 pathway has been shown to have anti-apoptotic effect during the process of reperfusion injury.^24,25^ It is not clear if melatonin maintains calcium homeostasis through modulating IP3R and SERCA2a via ERK1.

In the present study, the results showed that melatonin promote phosphorylation of ERK1 in cardiomyocytes against H/R, and pretreatment of PD98059 (ERK1 inhibitor) reduced phosphorylation of ERK1. In vitro results indicated melatonin prevents cardiomyocytes apoptosis against H/R. Meantime, melatonin can preserve structure of cardiomyocytes against reperfusion injury. Moreover, calcium overload induced by H/R is significantly reversed by melatonin. Moreover, the pretreatment of PD98059 inhibited the effect of melatonin on apoptosis, F-actin organization and calcium overload in cardiomyocytes against H/R. To further elucidate the underlying mechanism for protective effect of melatonin cardiomyocytes against H/R, we observed the effects of melatonin on expression of IP3R and SERCA2a. The results showed SERCA2a expression is decreased in H/R group compared with control group, but melatonin promoted SERCA2a expression in cardiomyocytes. Contrarily, H/R induces IP3R expression, and melatonin inhibits the expression of IP3R. Pretreatment of PD98059 reversed the effect of melatonin on expression of IP3R and SERCA2a. In vivo, myocardial IP3R level is reduced and SERCA2a expression is increased by pretreatment of melatonin, however, PD98059 reversed the effect of melatonin on expression of IP3R and SERCA2a. Melatonin in the dose used in the study did not show obvious side effects compared with other groups. In vivo results further confirmed that melatonin regulates the expression of IP3R and SERCA2a via ERK1. From the above results, it is reasonable to infer that melatonin could protect cardiomyocytes against reperfusion injury through affecting IP3R and SERCA2a expression to inhibit calcium overload via ERK1 pathway.

## Conclusion

Melatonin can protect cardiomyocytes against reperfusion injury through modulation of IP3R and SERCA2a attenuating calcium overload via ERK1 pathway. Improved calcium homeostasis followed by preserved function and structure of cardiomyocytes can decrease cardiomyocytes apoptosis and improve heart function. The present study provide more evidence for the use of melatonin to protect cardiac function in patients with STEMI undergoing myocardial reperfusion therapy.
